# A pyroptosis-related gene signature predicts prognosis and immune microenvironment in hepatocellular carcinoma

**DOI:** 10.1186/s12957-022-02617-y

**Published:** 2022-06-03

**Authors:** Yifeng Jin, Xiaofan Pu, Dongnan Ping, Chaojie Huang, Guoping Ding, Liping Cao

**Affiliations:** Department of General Surgery, Sir Run Run Shaw Hospital, School of Medicine, Zhejiang University, Hangzhou, China

**Keywords:** HCC, Pyroptosis, Prognosis, Immune checkpoint gene, Immune infiltrates

## Abstract

**Background:**

Hepatocellular carcinoma (HCC) is a highly malignant tumor with a very poor prognosis. Pyroptosis is an inflammatory form of cell death and plays an important role in cancer development. The prognostic value of pyroptosis-related genes (PRGs) in HCC has not been studied extensively.

**Methods:**

Unsupervised consensus clustering analysis was performed to identify two subtypes based on the expression profiles of prognostic PRGs in the The Cancer Genome Atlas (TCGA) database, and the differences between the two subtypes were compared. A prognostic model based on four PRGs was established by further least absolute shrinkage and selection operator (LASSO) Cox regression analysis and multivariate Cox regression analysis.

**Results:**

Two subtypes (clusters 1 and 2) were identified by consensus clustering based on prognostic PRGs in HCC. Survival outcomes, biological function, genomic alterations, immune cell infiltration, and immune checkpoint genes were compared between the subtypes. Cluster 2 had a worse survival outcome than cluster 1. Cluster 2 was enriched for hallmarks of cancer progression, TP53 mutation, tumor-promoting immune cells, and immune checkpoint genes, which may contribute to the poor prognosis. A prognostic risk signature that predicted the overall survival (OS) of patients was constructed and validated. Consequently, a risk score was calculated for each patient. Combined with the clinical characteristics, the risk score was found to be an independent prognostic factor for survival of HCC patients. Further analysis revealed that the risk score was closely associated with the levels of immune cell infiltration and the expression profiles of immune checkpoint genes.

**Conclusions:**

Collectively, our study established a prognostic risk signature for HCC and revealed a significant correlation between pyroptosis and the HCC immune microenvironment.

**Supplementary Information:**

The online version contains supplementary material available at 10.1186/s12957-022-02617-y.

## Introduction

Primary liver cancer is the sixth most frequently occurring malignancy and the third most common cause of cancer mortality worldwide [1]. Hepatocellular carcinoma (HCC) is the most dominant form of liver cancer and accounts for approximately 90% of cases [2]. Liver resection is the main curative treatment for HCC. However, most patients with HCC are diagnosed with intermediate or advanced stage disease, thus losing the opportunity for surgery. Even for patients who undergo surgical resection, postoperative recurrence or distant metastasis is common. Despite the significant progress made in diagnosis and treatment, the combination of the difficulty of early diagnosis and the ease of tumor recurrence and metastasis contribute to the poor prognosis of HCC. Therefore, prognostic biomarkers are urgently needed to help predict the outcomes for HCC patients and to guide clinical therapy.

Pyroptosis is an inflammatory form of programmed cell death, characterized by cellular swelling, many bubble-like protrusions, rupture, and release of inflammatory cytokines such as interleukin-1β (IL-1β) and and interleukin-18 (IL-18) [3]. Pyroptosis is mainly regulated by two main pathways: the caspase-1-mediated inflammasome pathway, involving the NLRP1, NLRP3, NLRC4, AIM2, and pyrin inflammasome (the canonical inflammasome pathway), and the caspase-4/5/11-mediated inflammasome pathway (the noncanonical inflammasome pathway) [4]. In both the canonical and noncanonical pathways, activated caspase cleaves pro-pyroptotic factor gasdermin D (GSDMD), leading to the generation of an N-terminal fragment (GSDMD-NT) [5]. The resultant N-terminal fragment binds to phosphatidylinositol phosphates and phosphatidylserine on the inner surface of cell membranes and oligomerizes to generate pores in the lipid bilayer [6]. These pores disrupt the osmotic balance within cells, resulting in rupture of the plasma membrane and the release of cell contents and pro-inflammatory cytokines [3].

Research has increasingly indicated that pyroptosis plays an important role in malignant cell transformation, growth, invasion, metastasis, and therapeutic responses. GSDMD and gasdermin E (GSDME) are two key effectors of pyroptosis. Wang et al. [7] reported that GSDMD expression was decreased in gastric cancers, and the downregulation of GSDMD could markedly promote the proliferation of tumors through cell cycle-related proteins accelerating S/G2 phase cell transition. GSDME can enhance immune responses to tumors such as triple-negative breast cancer by activating pyroptosis. Reduced GSDME expression is associated with decreased breast cancer survival, suggesting that GSDME might be a tumor suppressor in breast cancer [8]. One of the main characteristics of pyroptosis is the release of immunogenic cellular content and inflammatory cytokines, both of which are closely associated with the tumor microenvironment. Wang et al. [9] suggested that pyroptosis-induced inflammation triggers robust antitumor immunity and can synergize with the checkpoint blockade. All findings combined indicate that the role of pyroptosis in cancers varies among different tumor cells. In HCC, a recent study revealed a mechanism of action for sorafenib, eliciting macrophage pyroptosis that enhances NK-cell effector function and ultimately effective HCC cell killing [10]. Prognostic signatures based on PRGs were established for lung adenocarcinoma, ovarian cancer, and thyroid cancer [11–13]. However, the prognostic value of PRGs in HCC has not been studied extensively. In this study, we investigate the expression profiles of PRGs between normal liver tissue and HCC. Subsequently, clustering subtypes and risk models based on PRGs are established to improve prognostic risk stratification. The relationships between clustering subgroups, risk models, immune cell infiltration, and immune checkpoint genes are also analyzed to further explore the effect of PRGs on the tumor immune microenvironment (TIME).

## Materials and methods

### Data acquisition

The RNA-sequencing (RNA-seq) data of 371 HCC samples and 50 adjacent normal tissues, clinical characteristics, and survival information of liver hepatocellular carcinoma (LIHC) patients were obtained from the UCSC Xena database (https://xenabrowser.net/datapages/). Three-hundred sixty-four histologically diagnosed LIHC patients with both expression profiles and survival information were included for further analysis. The RNA-seq data and survival information of 233 HCC samples in ICGC-LIRI-JP (liver cancer, RIKEN, Japan) cohort as the external validation cohort were downloaded from the International Cancer Genome Consortium (ICGC) data portal (https://icgc.org/). All datasets used in this study are publicly available.

### Extraction of pyroptosis-related genes

A total of 30 pyroptosis-related genes were obtained from prior reviews [8, 12, 14–17], which are shown in Table S1. The expression differences of 30 PRGs between HCC cancer tissue and adjacent normal tissue were compared by the Wilcoxon rank-sum test. Univariate cox regression analysis was performed to evaluate the prognostic significance of the 30 PRGs.

### Consensus clustering

On the basis of the expression profiles of 11 prognostic PRGs, we clustered the patients into two subgroups by the R package “ConsensusClusterPlus” [18]. The clustering was performed using the following settings: 100 iterations, resample rate of 80%, K-means method, and Euclidean distance.

### Differential gene expression and functional enrichment analysis

The differentially expressed genes (DEGs) between the two clusters were identified using the R package “limma” [19]. Genes with a |log_2_ fold change (log_2_FC)| ≥ 1 and an adjusted *p*-values < 0.01 were considered as the selection criteria of DEGs. Gene ontology (GO) analysis and gene set enrichment analysis (GSEA) were carried out to functionally annotate genes that are differentially expressed in different clusters by using the R package “clusterProfiler” [20]. The hallmark gene sets retrieved from the molecular signatures database (https://www.gsea-msigdb.org/gsea/index.jsp) were used for the GSEA.

### Mutation analysis

Somatic mutation data (MuTect2 pipeline) of LIHC patients were downloaded using the “TCGAbiolinks” R package [21]. Afterward, the somatic mutation data of different clusters were analyzed, visualized, and compared by using the “maftools” R package [22]. TMB was defined as the number of somatic mutations in the coding region per megabase. TMB was compared using Wilcoxon rank-sum test between the two clusters.

### Establishment and validation of the PRG risk signature

LASSO Cox regression analysis was performed to eliminate 11 prognostic PRGs positively correlated with each other to avoid overfitting. Subsequently, stepwise multivariate regression analysis was conducted to further screen prognostic genes by using the lowest Akaike information criterions (AIC) value. Ultimately, a PRG risk signature involved four prognostic PRGs was established. The risk score was calculated as follows: risk score = coef (gene 1) × expr (gene 1) + coef (gene 2) × expr (gene 2) + coef (gene 3) × expr (gene 3) + coef (gene 4) × expr (gene 4), where “coef” indicates the coefficient of gene derived from the multivariate Cox analysis and “expr” is the gene expression level. The LIHC patients were classified into high-risk score group and low-risk score group based on median risk score as the cutoff value in TCGA group. We used Kaplan-Meier survival analysis to compare the survival outcomes between the high-risk and low-risk groups. The receiver operating characteristic curves (ROC) were utilized to assess the specificity and sensitivity of the model as well as the accuracy at 1, 2, and 3 years by “timeROC” package [23]. The accuracy of the PRG risk signature was then evaluated in the ICGC-LIRI-JP cohort.

### Correlation of PRG risk score with tumor immune infiltration levels

The immune score was calculated using the ESTIMATE algorithm through the R “estimate package” [24]. The immune cell infiltration was estimated by CIBERSORT algorithm [25]. The algorithm was based on leukocyte gene signature matrix (LM22) gene signature and 1000 permutations. Only samples with a *p*-value < 0.05 were included to perform the subsequent analysis. Correlation analysis between PRG risk score and the immune infiltration levels in HCC was conducted with Pearson correlation coefficients.

### Independence of the PRGs model

Multivariate and univariate Cox regression analysis were conducted to assess whether the risk score was an independent variable considering other clinical characteristics (age, gender, TNM stage, and grade) in the patients with HCC. A nomogram-integrated PRG risk score, age, TNM stage, and grade were established to predict the probable 1-, 2-, and 3-year survival of the patients. The concordance index (C-index) and calibration curves were applied to evaluate the concordance between predicted survival outcomes and practical outcomes.

### Statistical analysis

All statistical analysis was performed using the R software (R version 4.0.2). *P* < 0.05 represents statistical significance.

## Results

### Expression profile and prognostic value of PRGs in HCC

**Fig. 1:**
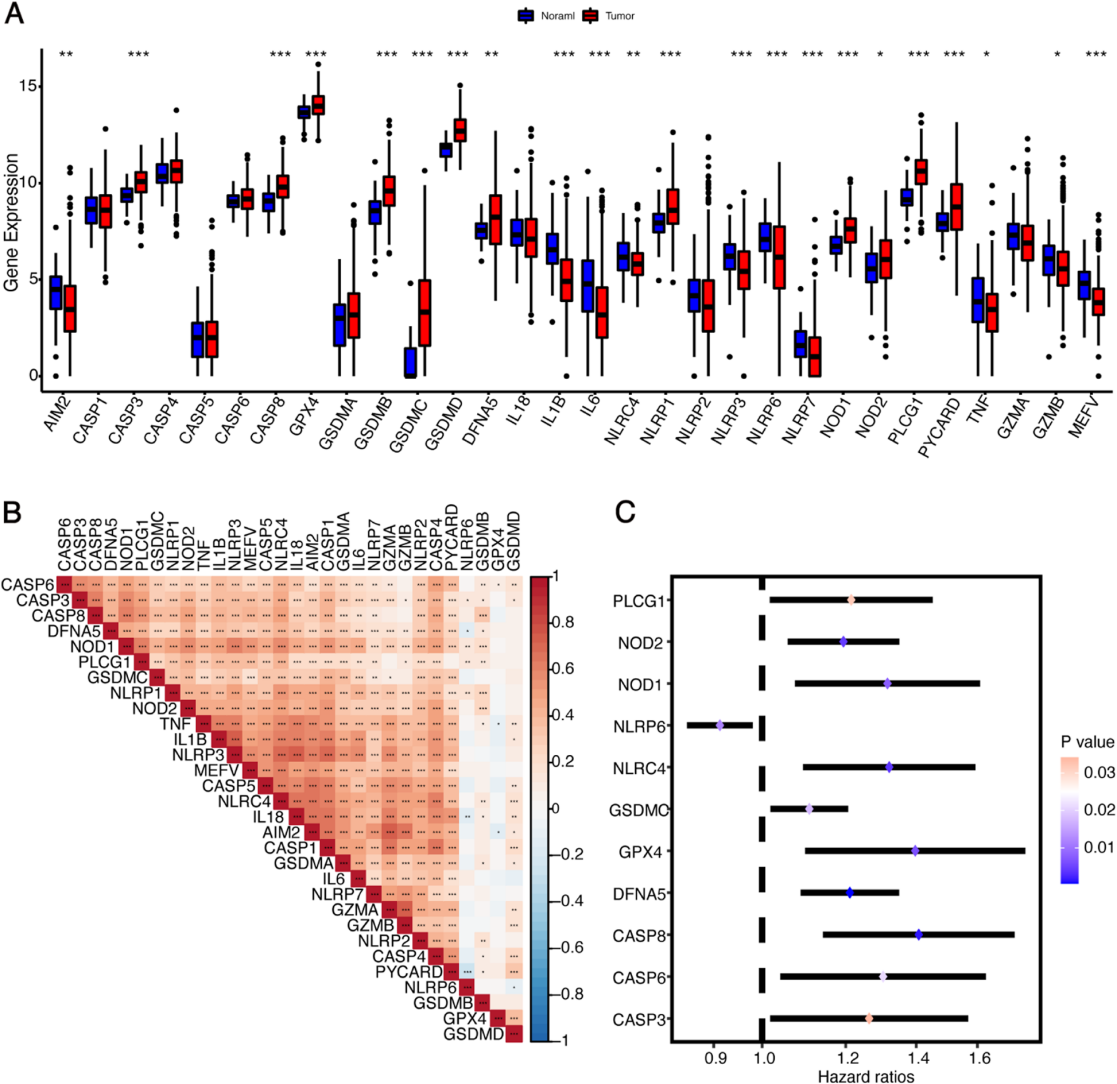
Expression landscape and prognostic value of PRGs in HCC. **A** The expressions of 30 pyroptosis-related genes (PRGs) in 50 normal liver tissues and 371 hepatocellular carcinoma tissues. **B** Spearman correlation analysis of the 30 PRGs in HCC. **C** Results of the univariate Cox regression analysis regarding OS in the TCGA cohort. *p < 0.05, **p < 0.01, and ***p < 0.001

We first compared the expression of the 30 PRGs in hepatocellular carcinoma tissues and normal liver tissues using The Cancer Genome Atlas Liver Hepatocellular Carcinoma (TCGA-LIHC) database. In total, 22 PRGs were differentially expressed between tumor tissues and adjacent nontumorous tissues. Among these genes, 10 genes (AIM2, IL1B, IL6, NLRC4, NLRP3, NLRP6, NLRP7, TNF, GZMB, and MEFV) were significantly downregulated, and 12 genes (CASP3, CASP8, GPX4, GSDMB, GSDMC, GSDMD, DFNA5, NLRP1, NOD1, NOD2, PLCG1, and PYCARD) were significantly upregulated in HCC tissues (Fig. 1A). The correlation matrix of the expression of the 30 PRGs is shown in Fig. 1B. Most of the genes were positively correlated with each other. We then explored the prognostic value of the 30 PRGs using univariate Cox regression analysis, and 11 of them (CASP3, CASP6, CASP8, GPX4, GSDMC, DFNA5, NLRC4, NLRP6, NOD1, NOD2, and PLCG1) were identified as significantly associated with the survival outcomes of LIHC patients (Fig. 1C). NLRP6 served as a protective factor for prognosis with a hazard ratios less than 1, whereas the rest of the prognostic PRGs was all risk factors with hazard ratios greater than 1. These results demonstrated that the expression profile of PRGs in HCC significantly differed from normal liver tissue, and that PRGs might play an important role in the prognosis of LIHC patients.

### LIHC classification pattern based on the prognostic PRGs

**Fig. 2:**
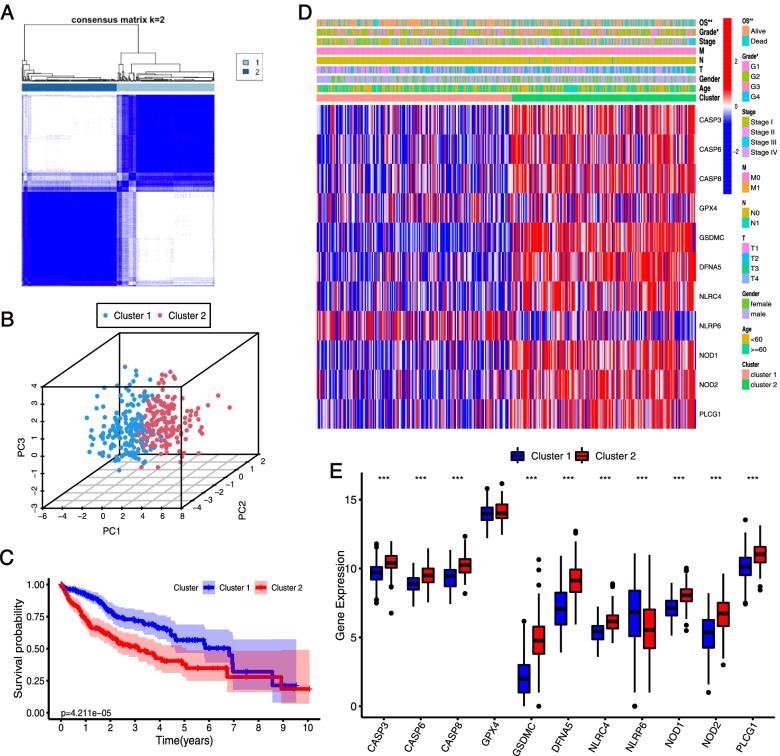
Identification of two subtypes of HCC in the TCGA cohort and differential characteristics between two subtypes. **A** Consensus clustering matrix for *k* = 2. **B** Principal component analysis of the gene-expression profiles in the TGGA HCC cohort. **C** Kaplan-Meier curves of overall survival (OS) for patients with HCC in clusters 1 and 2. **D** Heatmap and clinicopathologic characteristics of clusters 1 and 2. **E** The expressions of 11 prognostic PRGs in clusters 1 and 2. *p < 0.05, **p < 0.01, and ***p < 0.001

To explore the correlation between the expression of the 11 prognostic PRGs and LIHC subtypes, we performed an unsupervised consensus clustering analysis with all 364 LIHC patients. The clustering variable (*k*) = 2 was demonstrated to be the most appropriated selection form *k* = 2 to 10, and we divided the 364 LIHC patients into two clusters, namely cluster 1 (*n* = 185) and cluster 2 (*n* = 179) (Fig. 2A, Fig. SA–S1C). Principal component analysis (PCA) was conducted to test the difference between cluster 1 and cluster 2. Figure 2B shows that the two clusters were well separated from each other. The Kaplan-Meier curve indicated that patients in cluster 2 had a significantly worse OS (*p* < 0.001) than patients in cluster 1 (Fig. 2C). The clinicopathological features between the two clusters were then compared using the chi-squared test. Cluster 1 was associated with a low WHO grade (*p* < 0.05) and had a longer overall survival time (*p* < 0.01) compared with cluster 2 (Fig. 2D). The expression pattern of the 11 prognotic PRGs also varied significantly between the two clusters. Nine of the ten poor-prognostic genes (CASP3, CASP6, CASP8, GSDMC, DFNA5, NLRC4, NOD1, NOD2, and PLCG1) were enriched in cluster 2, while the only favorable-prognostic gene (NLRP6) was upregulated in cluster 1 (Fig. 2 D and E).

### Distinct immune cell infiltration and immune checkpoint

**Fig. 3:**
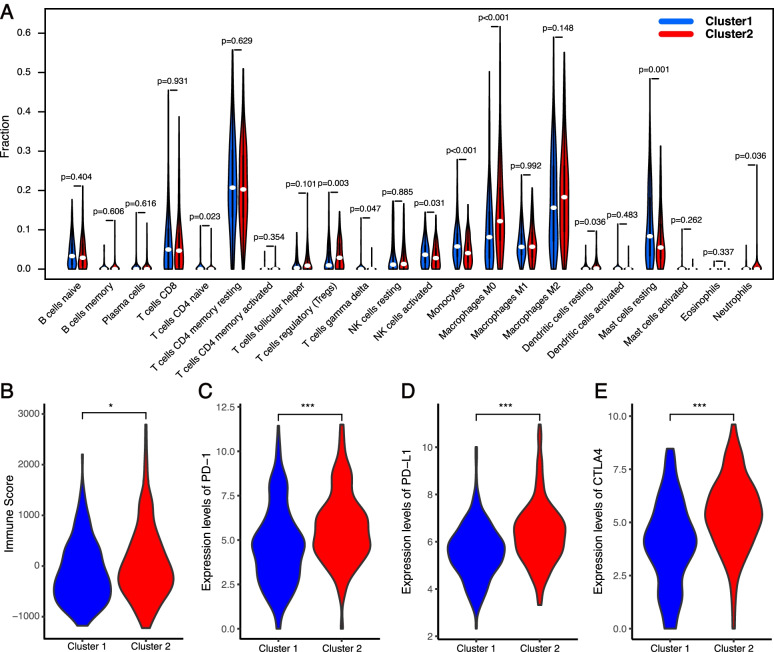
Landscape of immune cell infiltration and immune checkpoint genes in clusters 1 and 2. **A** The infiltration levels of 22 immune cell types in clusters 1 and 2. **B** Immunoscore in clusters 1 and 2. **C**–**E** The expressions of PD-1 (**C**), PD-L1 (**D**), and CTLA4 (**E**) in clusters 1 and 2. *p < 0.05, **p < 0.01, and ***p < 0.001

The immune microenvironment plays crucial roles in the pathogenesis of HCC [26]. Pyroptosis is a pro-inflammatory programmed cell death characterized by release of the immunogenic contents. We further explored the association of pyroptosis with the HCC immune microenvironment. The immune scores of the two clusters were estimated using the ESTIMATE algorithm. The immune score of cluster 2 was significantly higher than that of cluster 1 (Fig. 3B). Subsequently, the enrichment levels of 22 types of immune cells between cluster 1 and cluster 2 were compared. Cluster 1 showed higher infiltration levels of CD4 naive T cells, gamma delta T cells, activated NK cells, monocytes, and resting mast cells, whereas cluster 2 was more correlated with regulatory T cells (Tregs), M0 macrophages, M2 macrophages, resting dendritic cells, and neutrophils (Fig. 3A). The expression profiles of immune checkpoint genes, which play a key role in immune modulation, were also examined. The expression levels of PD-1, PD-L1, and CTLA-4 were all significantly upregulated in cluster 2 in comparison with cluster 1 (Fig. 3 C–E). Taken together, these results demonstrated that PRGs could indeed divided LIHC patients into two molecular subtypes with significantly different clinicopathological features, survival outcomes, and immune microenvironments.

### Functional analysis of DEGs

**Fig. 4:**
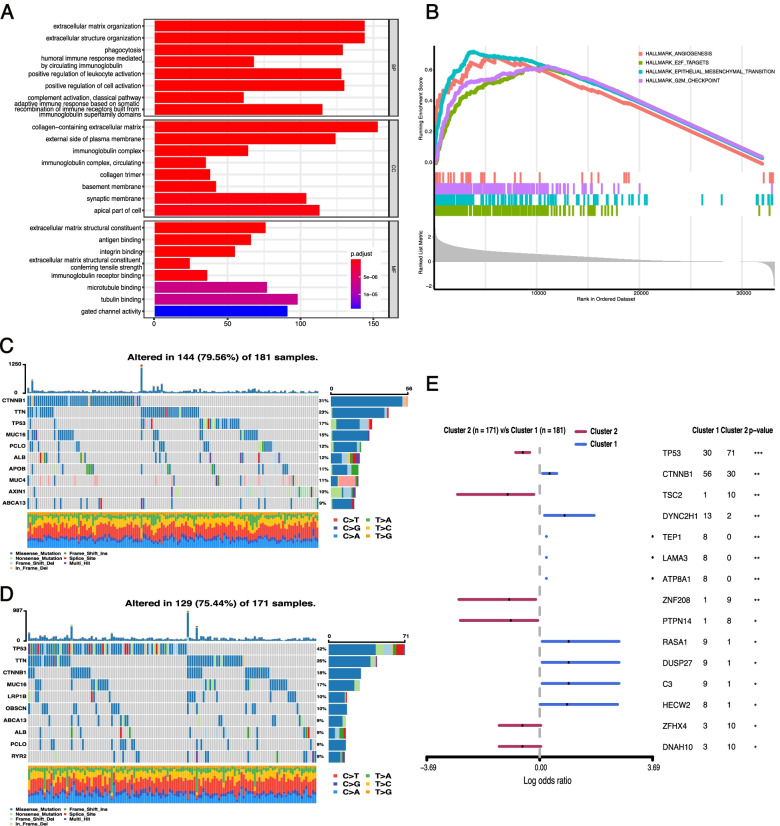
Functional analysis and somatic mutation characterization between two subtypes. **A** GO analysis of differentially expressed genes between clusters 1 and 2. **B** GSEA showed that hallmarks of cancer progression are differentially enriched in cluster 2. **C** and **D** Oncoplots of the top 10 mutated genes in clusters 1 (**C**) and 2 (**D**). **E** Forest plot of the differentially mutated genes between clusters 1 and 2. *p < 0.05, **p < 0.01, and ***p < 0.001

To better illuminate the underlying molecular differences between the two clusters, we identified the DEGs between cluster 1 and cluster 2 and annotated their functions using GO and GSEA analysis. With an adjusted cutoff value of *p* < 0.01 and |log_2_(fold change)| > 1, 3935 DEGs were identified. Among them, 3885 genes were upregulated, and 150 genes were downregulated in cluster 2 compared with cluster 1 (Fig. S2A). GO analysis indicated that the DEGs were related to terms including extracellular matrix organization, extracellular structure organization, positive regulation of cell activation, phagocytosis, and immune-related binding (Fig. 4A). The GSEA analysis showed that hallmarks of cancer progression—such as epithelial mesenchymal transition (normalized enrichment score [NES] = 1.704, adjusted *p*-values < 0.001), G2M checkpoint (normalized enrichment score [NES] = 1.478, adjusted *p*-values < 0.001), angiogenesis (normalized enrichment score [NES] = 1.513, adjusted *p*-values < 0.001), and E2F targets (normalized enrichment score [NES] = 1.457, adjusted i-values < 0.001)—were significantly enriched in cluster 2 (Fig. 4B).

### Genetic mutation landscapes of the two clusters

With rapid technological advances in genetics, the importance of the mutational profile of tumors, for both prognostic and predictive values, is being increasingly understood. We compared the tumor mutation burden (TMB) of the two clusters and found that the TMBs were not significantly different between the two clusters (Fig. S2B). Subsequently, the somatic mutation characterization of the two clusters was investigated. As shown in Fig. 4C, the top five genes with the highest mutation frequencies in cluster 1 were CTNNB1 (31%), TTN (23%), TP53 (17%), MUC16(15%), and ALB (12%), while those in cluster 2 (Fig. 4D) were TP53 (42%), TTN (26%), CTNNB1 (18%), MUC16 (17%), and LRP1B (10%). CTNNB1, DYNC2H1, TEP1, LAMA3, ATP8A1, RASA1, DUSP27, C3, and HECW2 were found to be highly mutated in cluster 1 compared with cluster 2, while TP53, TSC2, ZNF208, PTPN14, ZFHX4, and DNAH10 were found to be highly mutated in cluster 2 (Fig. 4E). These above functional enrichment analysis and mutational profile analysis indicated that the two clusters based on PRGs were also distinctively different in molecular basis and mutation characterization, which in turn affected their clinical characteristics and outcomes.

### Construction and validation of a risk model based on prognostic PRGs

**Fig. 5:**
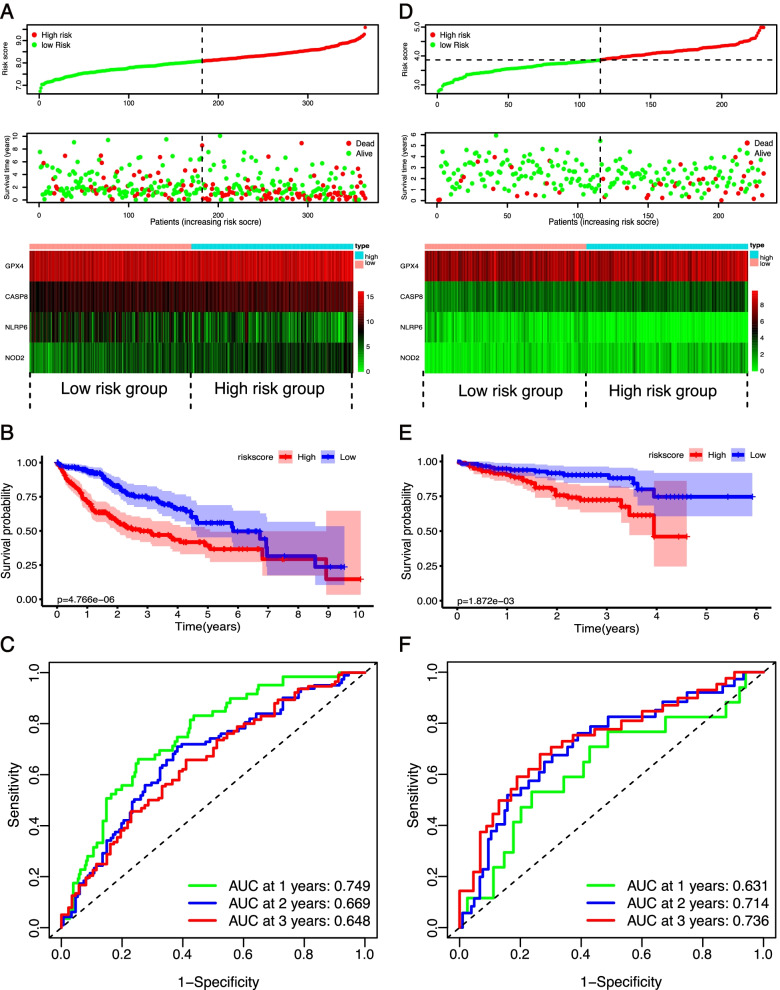
Construction and validation of prognostic PRG signature. **A** and **D** Distribution of risk score, OS, OS status, and heatmap of the four prognostic PRGs in the TCGA group (**A**) and ICGC group (**D**). **B** and **E** Kaplan-Meier curves of overall survival (OS) for patients with HCC divided by risk score in the TCGA group (**B**) and ICGC group (**E**). **C** and **F** Time-dependent ROC curves measuring the predictive value of the risk score in the TCGA group (**C**) and ICGC group (**F**)

To construct a prognostic gene model, 11 prognostic PRGs identified by univariate Cox analysis were further filtered by LASSO Cox regression analysis and stepwise multivariate regression analysis (Fig. S3 A–C). Four prognostic PRGs—GPX4, NLRP6, NOD2, and CASP8—were identified. A prognostic model was established to evaluate the survival risk for each patient as follows: PRG risk score = 0.3570335 × expression of GPX4 + (−0.1181711) × expression of NLRP6 + 0.1128816 × expression of NOD2 + 0.3197166 × expression of CSAP8. Afterwards, 364 LIHC patients were divided into high- and low-risk groups based on the median PRG risk score. We then investigated the correlation between the cluster groups and risk groups. As shown in Fig. S3D, the high-risk group was made up of 145 (79.67%) cluster 2 patients and 37 (20.33%) cluster 1 patients, whereas the low-risk group included 137 (75.27%) patients in cluster 1 and 45 (24.73%) patients in cluster 2. This result indicated that the risk groups dichotomized based on PRG risk score were largely consistent with clustering subgroups based on the expression of the 11 prognostic PRGs. The distribution of the risk scores, OS, OS status, and expression profiles of the four PRGs-based signatures is displayed in Fig. 5A. As shown in Fig. 5 A and B, patients in the high-risk group exhibited shorter OS times (*p* < 0.001) and higher mortality. The heatmap (Fig. 5A) and boxplot (Fig. S4A) indicated that risky PRGs, including GPX4, NOD2, and SCAF11, were highly enriched in the high-risk group, whereas the expression levels of the protective gene NLRP6 were significantly enriched in the low-risk group. We also performed ROC curve analysis to evaluate the predictive accuracy of our risk model. In the TCGC database, the AUCs for the PRG signatures at the 1-, 2-, and 3-year OS were 0.749, 0.669, and 0.648, respectively (Fig. 5C). We then verified our prognostic model using an independent verification dataset from the ICGC database. The risk score for each of the 232 LIHC patients from the ICGC was calculated using the same formula in the TCGA dataset, and patients were divided into high- and low-risk groups based on the median risk score. A similar result was observed between the ICGC and TCGA cohorts, where the high-risk group exhibited shorter OS times (*p* = 0.0018) and higher mortality (Fig. 5 D and E). The three risky PRGs and one protective PRG were also significantly enriched in the high-risk group and low-risk group, respectively (Fig. 5D and Fig. S4B). In the ICGC cohort, the AUCs of the PRG signature corresponding to 1, 2, and 3 years of survival were 0.631, 0.714, and 0.736, respectively (Fig. 5F). Taken together, our prognostic model showed a relatively high accuracy in predicting the prognosis of LIHC patients.

### The risk signature correlated with the clinical characteristics and the immune landscape of HCC

**Fig. 6:**
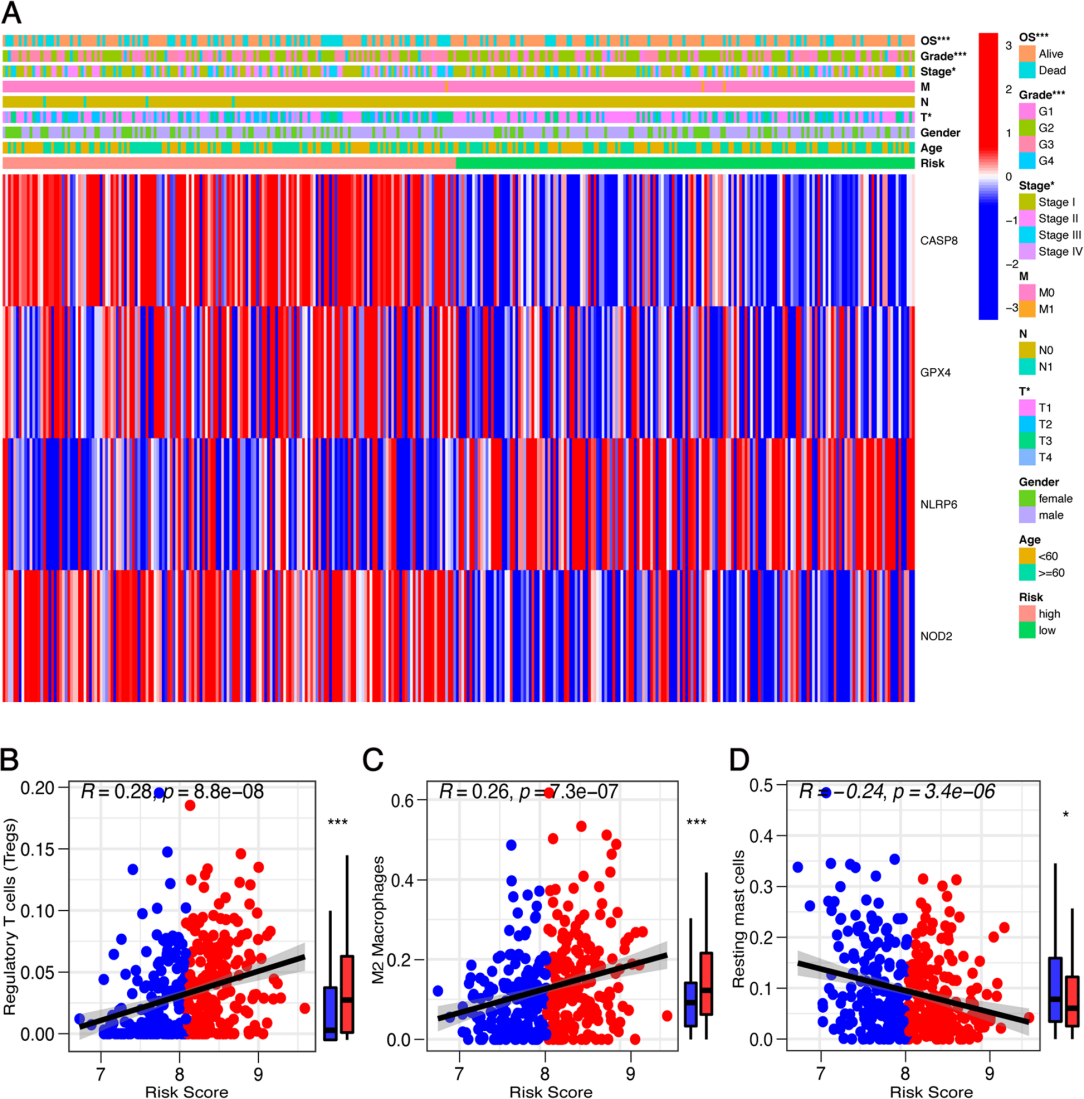
Prognostic risk scores correlated with clinicopathological characteristics and immune cell infiltration. **A** Heatmap and clinicopathologic characteristics of high- and low-risk groups. **B**–**D** correlation between the PRGs risk score and infiltrating levels of T cells regulatory (Tregs) (**B**), macrophages M0 (**C**), and mast cells resting (**D**). *p < 0.05, **p < 0.01, and ***p < 0.001

**Fig. 7:**
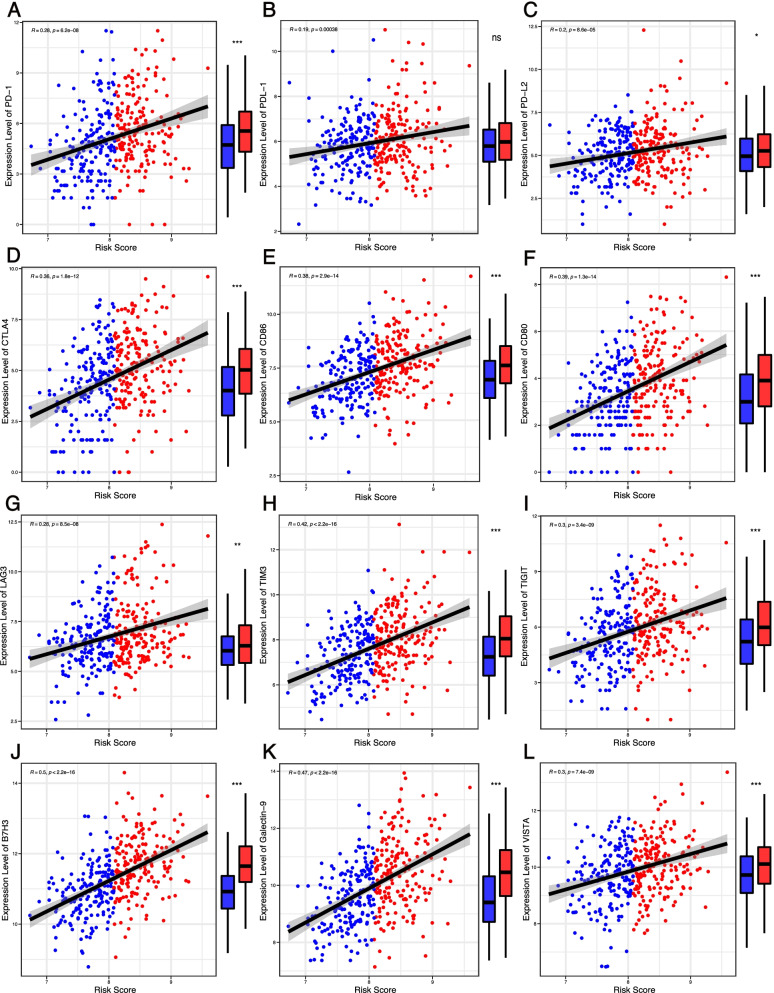
Prognostic risk scores correlated with expression levels of immune checkpoint genes. **A**–**L**: PD-1 (**A**), PD-L1 (**B**), PD-L2 (**C**), CTLA-4 (**D**), CD86 (**E**), CD80 (**F**), LAG-3 (**G**), TIM-3 (**H**), TIGIT (**I**), B7H3 (**J**), Galectin-9 (**K**), and VISTA (**L**). *p < 0.05, **p < 0.01, and ***p < 0.001

**Fig. 8:**
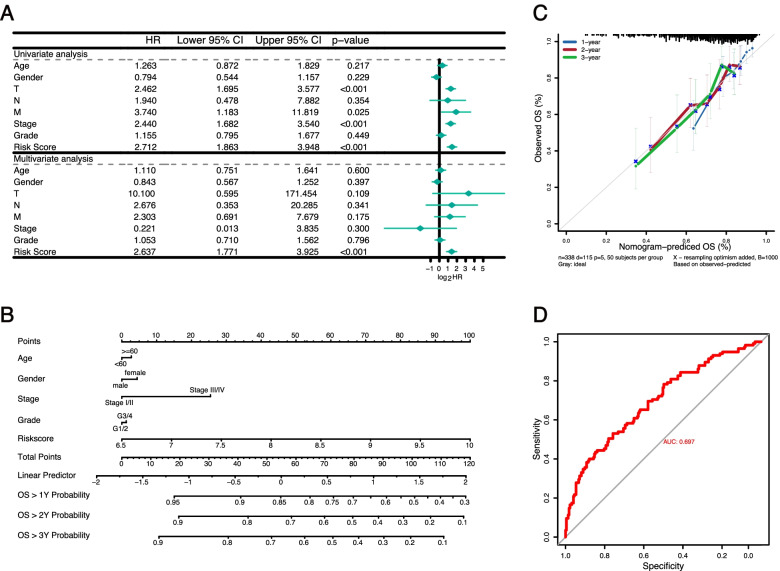
Construction of a predictive nomogram. **A** Univariate and multivariate Cox regression of OS for clinical characteristics and PRG risk score in HCC. **B** Nomogram to predict the 1-year, 2-year, and 3-year overall survival rate of LIHC patients. **C** Calibration curves of the nomogram for predicting the probability of OS at 1, 2, and 3 years. **D** ROC curves of the nomogram

We evaluated the relationship between the PRG risk scores and clinical characteristics. As shown in the heatmap, the difference in T stage (*p* < 0.05), TNM stage (*p* < 0.05), grade (*p* < 0.001), and overall survival (*p* < 0.001) between the high- and low-risk groups was significant (Fig. 6A). Tumor-infiltrating immune cells (TIICs) play essential roles in cancer development and are closely associated with clinical outcomes. We assessed the proportions of different TIICs and explored the correlation between the PRG risk score and TIICs. Figure 6 B–D demonstrates that the PRG risk score was positively correlated with the infiltration levels of Tregs and M2 macrophages but negatively correlated with the infiltration levels of resting mast cells. Moreover, we investigated the relationship between the PRG risk score and the expression of immune checkpoints. Figure 7 A–F shows that the PRG risk score had a significant positive correlation with the expression of common immune checkpoints and their ligands, namely anti-programmed cell death protein 1 (PD-1) and its ligand programmed cell death ligand 1/2 (PD-L1/2) and anti-cytotoxic T-lymphocyte-associated antigen-4 (CTLA-4) and its ligand CD86/CD80. The expression of novel immune checkpoints, including lymphocyte activation gene-3 (LAG-3), T-cell immunoglobulin and mucin-domain containing-3 (TIM-3), T-cell immunoglobulin and ITIM domain (TIGIT), B7H3, Galectin-9, and V-domain Ig suppressor of T cell activation (VISTA), was also significantly associated with the PRG risk score (Fig. 8G–L). Taken together, these results suggested that the PRG risk score is significantly associated with the clinical characteristics, the landscape of immune cell infiltrations, and the expression levels of immune checkpoint genes in LIHC patients.

### The risk signature is an independent prognostic factor for patients with HCC

Univariate and multivariate Cox regression analyses were carried out to assess whether the risk signature was an independent prognostic predictor for OS in the TCGA cohort. As described in Fig. 8A, the results of the univariate Cox regression analysis showed that T stage (*HR*: 2.462, 95% *CI* :1.695–3.577, *p* < 0.001), M stage (*HR*: 3.740, 95% *CI*: 1.183–11.819, *p* = 0.025), TNM stage (*HR*: 2.440, 95% *CI*: 1.682–3.540, *p* < 0.001), and the PRG risk score (*HR*: 2.712, 95% *CI*:1.863–3.948, *p* < 0.001) were significantly associated with the prognosis of LIHC patients. After multivariate Cox regression analysis, only the PRG risk score (*HR*: 2.637, 95% *CI*: 1.771–3.925, *p* < 0.001) remained an independent predictor for the prognosis of LIHC patients. To facilitate the use of our PRGs prognostic model, we established a nomogram comprising the PRG risk score and clinical characteristics for predicting LIHC prognosis. By comparison with clinical characteristics, the PRG risk score showed predominant predictive ability in the nomogram (Fig. 8B). A calibration plot demonstrated ideal consistency compared with the ideal model, indicating that the nomogram has stability for predicting LIHC patient prognosis in clinical practice (Fig. 8C). The C-index of the established nomogram for OS prediction was 0.695 (Fig. 8D). These results collectively verified that our PRG risk signature could reliably serve as an independent prognostic factor for LIHC patients.

## Discussion

Pyroptosis is a novel form of programmed cell death triggered by certain inflammasomes. Studies have found that pyroptosis plays a dual role in the proliferation, invasion, and metastasis of tumors. On the one hand, pyroptosis can promote inflammatory cell death of cancer and inhibit the proliferation and migration of cancer cells. On the other hand, inflammatory mediators IL-1 and IL-18, released by the activation of pyroptosis, can promote the progression of various cancers [27]. The expression level of GSDME was significantly increased in esophageal squamous cell carcinoma (ESCC) tissues than in normal esophageal tissues and was significantly correlated with a better prognosis in patients with ESCC. In GSDME high-expressed ESCC cell lines, the serine/threonine protein kinase PLK1 inhibitor BI2536 combined with cisplatin can increase chemosensitivity by inducing GSDMD-mediated pyroptosis [28]. The expression of GSDMD was significantly upregulated in non-small cell lung cancer (NSCLC), and higher GSDMD expression is associated with invasive features, including more advanced tumor-node-metastasis stages and larger tumor sizes [29]. Apoptosis, another form of caspase-mediated cell death, has been found to be extensively involved in the tumorigenesis and development of HCC. Inhibition of HIF1A-AS1, a long noncoding RNA (lncRNA) overexpressing in HCC tissues, could enhanced starvation-induced apoptosis in HCC cell [30]. A prognostic model involved apoptosis-related genes with impressive prognostic predictive power was also established in HCC [31]. Furthermore, numerous prognostic genes and prognostic signature for HCC have been reported by many studies [32–35] and further evaluated by some meta-analysis [36, 37]. However, the relationship between pyroptosis and HCC is not fully understood. Most studies regarding pyroptosis in human HCC have found pyroptosis plays an antitumor role in HCC. For example, Wei et al. [38] reported that the expression of NLRP3 was either completely lost or significantly downregulated in human HCC, and that the deficiency correlated significantly with poor pathological differentiation and advanced stages, indicating that the NLRP3 inflammasome was involved in the progression of HCC. One distinct characteristic which separates pyroptosis from other kinds of cell death is the activation of caspase-1. Chu et al. [39] observed a significantly decreased expression of caspase-1 in HCC tissues from patients and found that activation of caspase-1-dependent pyroptosis shows therapeutic potential against HCC. In this study, we first studied the mRNA levels of 30 PRGs in HCC samples and adjacent normal tissues and found that 22 PRGs were differentially expressed. In detail, the expression levels of AIM2, IL1B, IL6, NLRC4, NLRP3, NLRP6, NLRP7, TNF, GZMB, and MEFV were decreased, while the expression levels of CASP3, CASP8, GPX4, GSDMB, GSDMC, GSDMD, DFNA5, NLRP1, NOD1, NOD2, PLCG1, and PYCARD were increased in HCC samples compared with normal tissues. These results indicated that pyroptosis might be extensively involved in HCC tumorigenesis. Univariate Cox regression analysis was performed to assess the prognostic value of the 30 PRGs and found that 11 PRGs were significantly associated with overall survival. Based on the expression profiles of the 11 prognostic genes, LIHC patients were classified into two clusters with distinct survival outcomes and clinicopathological features. Patients in clusters 2 were more likely to have a shorter survival time, higher WHO grade, and higher immune score. Further analysis revealed that cluster 2 had increased levels of immune checkpoint genes. The CIBERSORT algorithm was used to calculate the proportion of different types of tumor-infiltrating immune cells. The result showed that compared with cluster 1, immune cells that promote tumor proliferation, therapeutic resistance, and metastasis, such as M2 macrophages, regulatory T cells, and neutrophils, were enriched in cluster 2 [40–42]. These results suggest a significant correlation between PRGs and tumor-immune infiltration.

Certain gene mutations in HCC are closely related to the survival outcomes and vary significantly between different subgroups of HCC. TERT promoter mutations, the tumor protein p53 (TP53), and WNT pathway oncogene catenin beta 1 (CTNNB1) are the most frequent somatic genetic alterations in HCC [43]. Several studies have shown that HCCs with mutations in CTNNB1 display a particular phenotype with well-differentiated tumors and better prognostic outcomes. HCCs with TP53 mutation and an absence of CTNNB1 mutation display aggressive tumor characteristics and worse prognosis [44]. We found that our subgroups based on PRGs had significantly different mutational profiles. In cluster 1, which had a longer overall survival time, 31% of patients had mutations in CTNNB1, and 17% had mutations in TP53. In cluster 2, which had worse prognosis, 42% of patients had mutations in TP53, and 18% of patients had mutations in CTNNB1.

Our study constructed a prognostic gene model based on four prognostic PRGs (GPX4, NLRP6, NOD2, and CASP8) and generated a PRG risk score for each LIHC patient. Glutathione peroxidase 4 (GPX4) is an essential regulator of ferroptosis, and an antitumor effect of GPX4 inhibition-induced ferroptosis has been found in a variety of cancers, such as breast, kidney, and colorectal cancers [45–47]. Recent studies have suggested that GPX4 also functions as an important gateway to pyroptosis. Kang et al. [48] found that GPX4 is upregulated in innate immune cells to counter-regulate GSDMD-N-mediated pyroptotic cell death, thereby preventing lethal systemic inflammation. Guerriero E. reported a significantly overexpression of GPX4 in HCC compared with nontumor tissues, and this overexpression was associated with an increased malignancy grade [49]. However, another study found that GPX4 suppressed the formation and progression of HCC by inhibition of angiogenesis and tumor cell proliferation as well as by immune-mediated mechanisms [50]. In our study, multivariate Cox regression analysis showed that GPX4 was a risky gene with an HR larger than 1 (*HR* = 1.45, 95% *CI* = 1.14–1.85), and GPX4 expression levels were significantly upregulated in the high-risk group compared with those in the low-risk group. NLR family pyrin domain containing 6 (NLRP6) is a member of the NLR (nucleotide-oligomerization domain-like receptor) family that patrols the cytosolic compartment of cells to detect pathogen- and damage-associated molecular patterns [51]. Wang et al. [52] demonstrated that NLRP6 was downregulated in approximately 75% of primary gastric cancer cases and functioned as a negative regulator of gastric cancer. In another study, NLRP6 was found to perform essential functions in the regulation of tissue repair necessary for protection against chemically induced injury [53]. Several studies have suggested a protective role of NLRP6 in a variety of liver diseases, such as liver cirrhosis, liver injury, acute liver failure, and alcoholic hepatitis [54–57]. However, the relationships between NLRP6 and HCC remain largely unknown. In our study, we found that NLRP6 was one of the pyroptosis-related prognostic biomarkers in HCC and was likely to serve as a protective factor against HCC. Further in vivo and in vitro studies should be performed to elucidate the role of NLRP6 in HCC. Nucleotide-binding oligomerization domain 2 (NOD2) is a member of the family of pattern recognition receptors (PRRs) that can initiate potent immune response against pathogens [58]. Ma et al. [59] reported that NOD2 deficiency promoted hepatocarcinogenesis, while overexpression of NOD2 in HCC cells inhibited tumorigenesis and reversed resistance to chemotherapy. However, a recent study by Zhou et al. [60] suggested an opposite role for NOD2 in the development of HCC. Zhou et al. showed that NOD2 was overexpressed in HCC samples and closely correlated with poor prognosis of LIHC patients. Loss of hepatic NOD2 attenuated the tumorigenesis of DEN/CCl4-induced HCC in hepatocyte-specific Nod2-knockout mice. We found that NOD2 was a cancer-promoting gene, as it was enriched in the high-risk group and associated with poor prognosis among LIHC patients. Given these contradictory results, further studies will be needed to understand the relationship between NOD2 and the development of HCC. Caspase-8 (CASP8) has long been considered as an initiator of the extrinsic apoptotic pathway [61]. Recent studies have suggested that CASP8 also cleaves and activates GSDMD, inducing pyroptosis in response to activation of the extrinsic apoptosis signaling pathway [62]. An increasing number of studies have confirmed that CASP8 is associated with tumor growth and invasion, angiogenesis, metastasis, therapeutic resistance, and poor clinical outcomes [63, 64]. In our research, we found that CASP8 was upregulated in the high-risk group and was prognostic for poor survival.

The prognostic value of the PRG risk model was evaluated in patients with LIHC and validated in the external ICGC cohort. The risk score obtained from the risk signatures effectively classified the patients with LIHC into high- and low-risk groups. The OS of the patients in the high-risk group was shorter than that of the patients in the low-risk group in the TCGA cohort. Consistent results were also obtained in an independent ICGC validation cohort. The risk groups classified by the risk score were largely consistent with the classification based on the expression profile of prognostic PRGs. These results indicated that the risk signature based on PRGs may help to improve the clinical assessment of patients, optimize medical intervention, and identify potential therapeutic targets for different subgroups.

Accumulating evidence has revealed that immune infiltrates in tumor microenvironment play a significant role in the prognosis of HCC. SNRPC, PKB, and DCK have been discovered to significantly correlate with patient outcomes and immune cell infiltration in hepatocellular carcinoma [65–67]. We further explored the relationship between the PRG risk score and tumor-infiltrating immune cells. The PRG risk score was positively correlated with Tregs and M2 macrophages but negatively correlated with the infiltration levels of resting mast cells. Notably, the expression levels of several immune checkpoint-related genes, including PD-1, PD-L1, PD-L2, CTLA-4, CD86, CD80, LAG-3, TIM-3, TIGIT, B7H3, Galectin-9, and VISTA, were all significantly enriched in the high-risk group and correlated with the risk score. Taken together, we speculated that overexpression of immune checkpoint and tumor-promoting immune cells impaired the efficacy of the antitumor immune response, thus leading to the unfavorable prognosis in the high-risk group. Immunotherapy is a promising treatment option for HCC, and its efficacy is significantly influenced by the HCC immune microenvironment [68]. Given its close association with the HCC immune microenvironment, our PRG risk signature may serve as a useful tool to stratify LIHC patients into different immune subtypes and predict the sensitivity to immunotherapy.

Univariate and multivariate Cox regression analysis showed that the risk score was an independent prognostic factor for LIHC patients. We integrated risk score, TNM stage, grade, age, and gender to construct a nomogram, and we found a good agreement between the actual OS and predicted OS at 1, 2, and 3 years. These results indicated that the PRG signature combined with commonly available clinical characteristics might be a promising prognostic tool for LIHC patients.

Our study has a few limitations. First, our study is based on the TCGA public database and only verified by the ICGC public database. Second, given the high heterogeneity of HCC, relatively few tumor samples are included in our research. Furthermore, only protein-coding genes were explored in the present study. However, it has been established that noncoding RNAs, such as lncRNA and microRNAs, also play essential role in tumorigenesis, progression, and immunity of HCC. For example, Xue et al. reported that lncRNA ZEB1-AS1 inhibited HCC progression through miR-23c [69]. In another study, a prognostic signature and a nomogram integrating three lncRNAs were constructed for HCC patients [70]. Further studies that integrated multicenter datasets and different types of RNA genes are needed to test the results. Thirdly, our research should be further explored by both in vitro and in vivo experiments to elucidate the precise roles of pyroptosis in HCC.

In conclusion, our study showed that pyroptosis is heavily involved in HCC. Most of the PRGs were differently expressed between normal and HCC tissues. Further comprehensive bioinformatics analysis identified HCC subtypes based on PRG signatures. The survival outcomes, clinical characteristics, genomic alterations, and immune microenvironment differed significantly between the subgroups. The risk score generated by the PRG signature is closely correlated with the infiltration levels of immune cells and the expression profile of immune checkpoint genes.

## Supplementary Information

**Additional file 1: Figure S1.** Consensus clusters by PRGs in TCGA cohort. **Figure S2.** DEGs and TMB scores of the clusters 1 and 2. **Figure S3.** Screening of four PRGs signature genes. **Figure S4.** The expressions of four prognostic PRGs in high- and low-risk groups. **Table S1.** Names of 30 pyroptosis-related genes.

## Data Availability

Gene expression profiles, clinical information, and mutation data of HCC in this study are available from the public database (TCGA, https://xenabrowser.net/datapages/; ICGC, https://icgc.org/).
