# Supplementary material for: A pyroptosis-related gene signature predicts prognosis and immune microenvironment in hepatocellular carcinoma
Source: World J Surg Oncol. 2022 Jun 3;20:179. doi: 10.1186/s12957-022-02617-y (PMC9164458; doi:10.1186/s12957-022-02617-y)
Supplement: Supplementary file 1 — Additional file 1: Figure S1. Consensus clusters by PRGs in TCGA cohort. Figure S2. DEGs and TMB scores of the clusters 1 and 2. Figure S3. Screening of four PRGs signature genes. Figure S4. The expressions of four prognostic PRGs in high- and low-risk groups. Table S1. Names of 30 pyroptosis-related genes. [file 12957_2022_2617_MOESM1_ESM.pdf]

Figure S1. Consensus clusters by PRGs in TCGA cohort.

(A) Consensus clustering cumulative distribution function (CDF) for k=2-9. (B) Relative change in area under the CDF curve for k = 2-9. (C) Tracking plot for k=2-9.

Figure S2. DEGs and TMB scores of the clusters 1 and 2.

(A) Volcano plot of DEGs in the cluster 2 when compared with the cluster 1. (B) TMB scores in the clusters 1 and 2.

Figure S3. Screening of four PRGs signature genes.

(A) Partial likelihood deviance for tuning the parameter selection in the LASSO regression model. (B) LASSO coefficient profiles of the 11 prognostic PRGs. (C) Hazard ratios and corresponding 95% confidence intervals calculated by stepwise multivariate regression analysis. (D) barplot of distribution of cluster 1/2 by risk subgroups. *p < 0.05, **p < 0.01, and ***p < 0.001.

Figure S4. The expressions of four prognostic PRGs in high- and low-risk groups.

(A and B) The expressions of four prognostic PRGs in high- and low-risk groups in the TCGA group (A) and ICGC group (B). *p < 0.05, **p < 0.01, and ***p < 0.001.

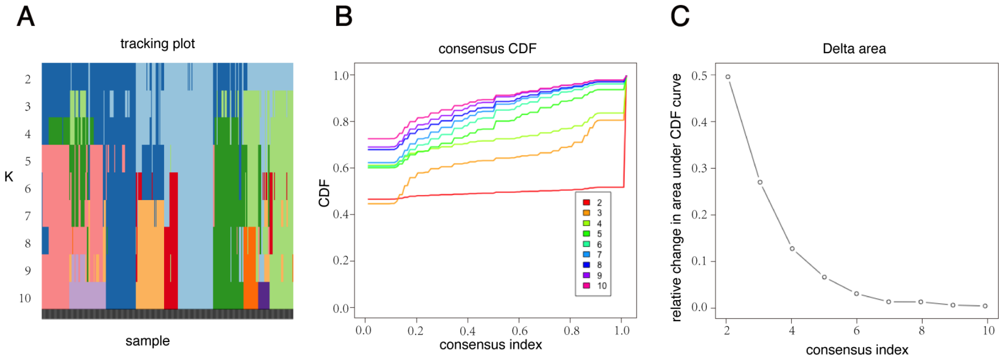

Figure S1. Consensus clusters by PRGs in TCGA cohort

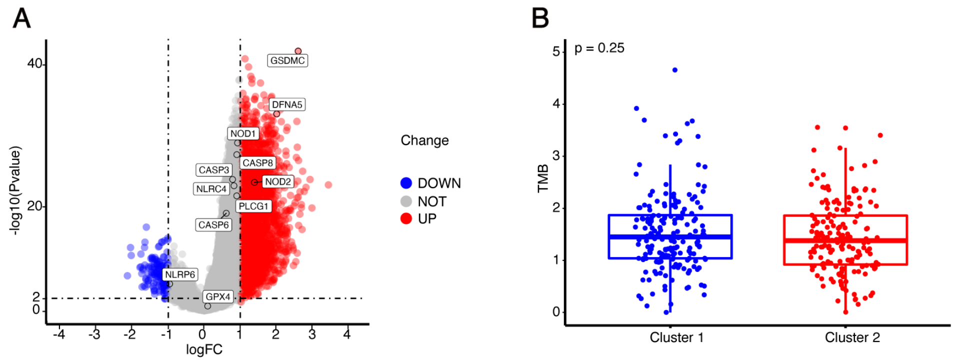

Figure S2. DEGs and TMB scores of clusters 1 and 2

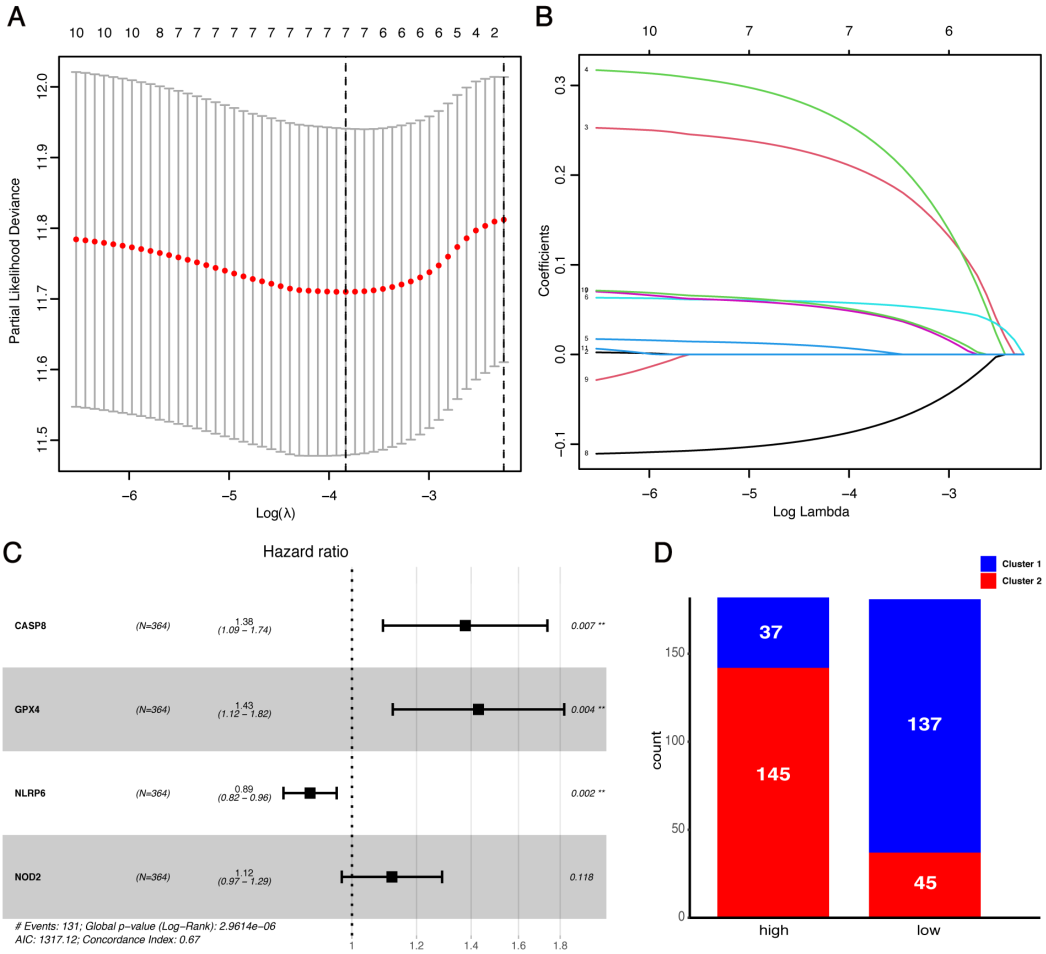

Figure S3. Screening of four PRGs signature genes

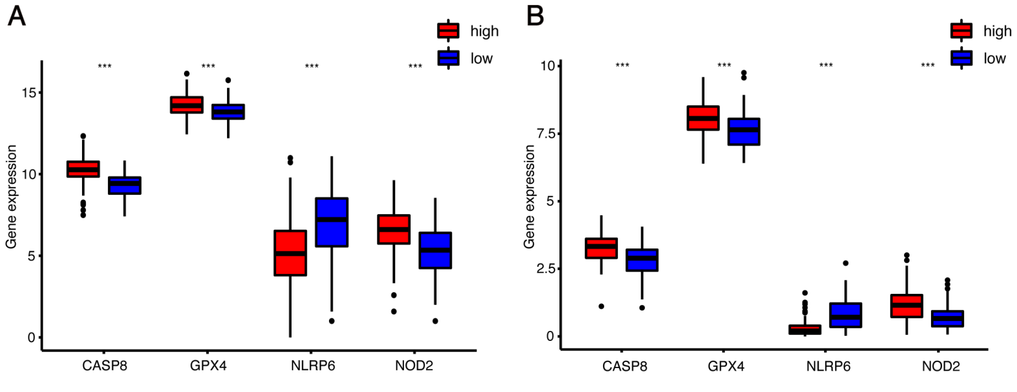

Figure S4. The expressions of four prognostic PRGs in high- and low-risk groups

Table S1. Names of 30 pyroptosis-related genes.

**Genes**	**Full-names**
AIM2	Absent in melanoma 2
CASP1	cysteine-aspartic acid protease-1
CASP3	cysteine-aspartic acid protease-3
CASP4	cysteine-aspartic acid protease-4
CASP5	cysteine-aspartic acid protease-5
CASP6	cysteine-aspartic acid protease-6
CASP8	cysteine-aspartic acid protease-8
GPX4	glutathione peroxidase 4
GSDMA	gasdermin A
GSDMB	gasdermin B
GSDMC	gasdermin C
GSDMD	gasdermin D
DFNA5	gasdermin E
IL18	interleukin 18
IL1B	interleukin 1 beta
IL6	interleukin 6
NLRC4	NLR family CARD domain containing 4
NLRP1	NLR family pyrin domain containing 1
NLRP2	NLR family pyrin domain containing 2
NLRP3	NLR family pyrin domain containing 3
NLRP6	NLR family pyrin domain containing 6
NLRP7	NLR family pyrin domain containing 7
NOD1	nucleotide binding oligomerization domain containing 1
NOD2	nucleotide binding oligomerization domain containing 2
PLCG1	phospholipase C gamma 1
PYCARD	PYD and CARD domain containing
TNF	tumor necrosis factor
GZMA	Granzyme A
GZMB	Granzyme B
MEFV	Mediterranean Fever
